# Purinergic ATP triggers moxibustion-induced local anti-nociceptive effect on inflammatory pain model

**DOI:** 10.1007/s11302-021-09815-5

**Published:** 2021-08-11

**Authors:** Hai-Yan Yin, Ya-Peng Fan, Juan Liu, Dao-Tong Li, Jing Guo, Shu-Guang Yu

**Affiliations:** 1 School of Acupuncture and Tuina, Chengdu University of Traditional Chinese Medicine, Chengdu, 610075 China; 2 Acupuncture & Chronobiology Key Laboratory of Sichuan Province, Chengdu, 610075 China; 3 Luoyang Orthopedic-Traumatological Hospital of Henan Province, Luoyang, 471000 China

**Keywords:** ATP, Purinergic signalling, Inflammatory pain, Moxibustion therapy

## Abstract

Purinergic signalling adenosine and its A1 receptors have been demonstrated to get involved in the mechanism of acupuncture (needling therapy) analgesia. However, whether purinergic signalling would be responsible for the local analgesic effect of moxibustion therapy, the predominant member in acupuncture family procedures also could trigger analgesic effect on pain diseases, it still remains unclear. In this study, we applied moxibustion to generate analgesic effect on complete Freund’s adjuvant (CFA)-induced inflammatory pain rats and detected the purine released from moxibustioned-acupoint by high-performance liquid chromatography (HPLC) approach. Intramuscular injection of ARL67156 into the acupoint Zusanli (ST36) to inhibit the breakdown of ATP showed the analgesic effect of moxibustion was increased while intramuscular injection of ATPase to speed up ATP hydrolysis caused a reduced moxibustion-induced analgesia. These data implied that purinergic ATP at the location of ST36 acupoint is a potentially beneficial factor for moxibustion-induced analgesia.

## Introduction

Since purinergic signalling was proposed by Prof. Geoffrey Burnstock (1929–2020) in 1972 [1], it has been recognized as one of promising drug target [2–8]. Purinergic signalling is also hypothesized and has been found to get involved in the mechanism of acupuncture therapy (i.e., the acupuncture family procedure, including needle therapy, moxibustion therapy, cupping therapy, etc.) and to address the potential mechanism of acupuncture-induced analgesia [9–13]. In previous studies, apart from adenosine (ADO) and A1 receptor that were identified to mediate local anti-nociceptive effect of acupuncture [14, 15], purinergic receptors A2a, P2X3, P2X4, and P2X7 have also been applied to punch the truth of acupuncture analgesia [16–22]. Moxibustion is one of important members in traditional Chinese medicine (TCM) therapies, which is recognized to share equal values with acupuncture in treating diseases in the traditional medical system of China [11]. During moxibustion intervention, the ignited mugwort (Artemisia vulgaris from traditional Chinese medicine) is applied directly or indirectly at acupuncture points or other specific parts of the body to cure or prevent from a variety of diseases, especially for chronic pain, such as visceral pain of primary dysmenorrhea [23], primary osteoporosis pain [24], knee osteoarthritis pain [25], etc. And many animal experiments also proved that administration of moxibustion raised analgesic effect [26–29]. Since acupuncture and moxibustion could induce anti-nociceptive effect by stimulating acupoints, and purinergic signalling in local acupoints has proved to be involved in acupuncture analgesia, here is a question: does purinergic signalling also play an important role in moxibustion-induced analgesia? It still remains unclear. Hence, this study is anticipated to test the hypothesis whether purinergic signalling take part in moxibustion-induced analgesia and provide more evidence to enrich the mechanism of acupuncture analgesia.

## Methods

### Inflammatory pain animal model

**Fig. 1:**
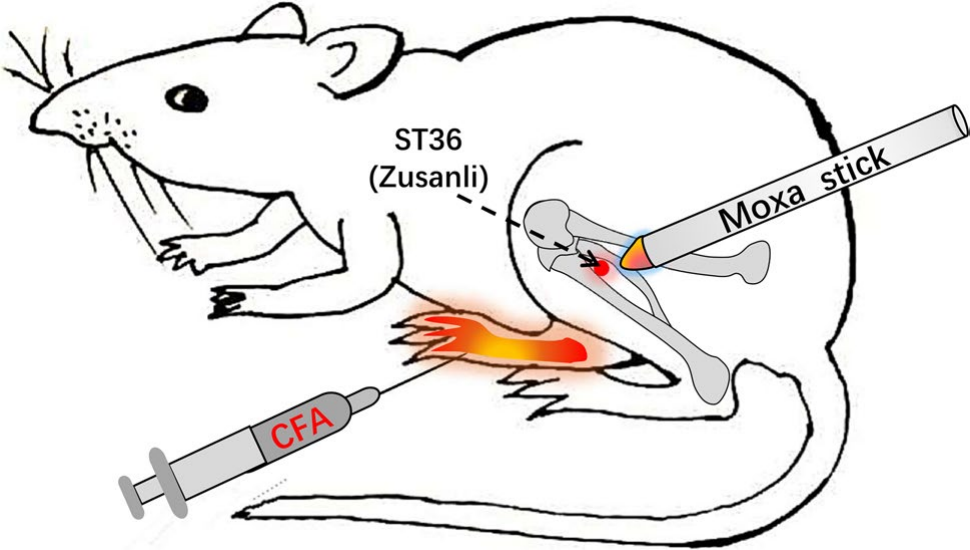
Schematic diagram inflammatory pain animal model and moxibustion intervention. The inflammatory-pain animal model was induced by injecting 100 μl of CFA into the right hind paw of rat. ST36 acupoint location: 5 mm below and lateral to the anterior tubercle of the tibia unilaterally. Moxibustion was applied at a distance of approximately 1 cm over the *Zusanli* (ST36) acupoint onto the right limb of the animals using moxa sticks

Adult female Sprague–Dawley rats weighing 200–220 g were used in this experiment. All rats were allowed to freely take food and water ad libitum and maintained in animal room of automatically controlled day cycles (12:12 = light: dark cycle) at 24 ± 2 °C. The animal model of inflammatory pain was induced by injecting 100 μl of complete Freund’s adjuvant (CFA, Sigma-Aldrich, St. Louis, MO) into the right hind paw of rats (Fig. 1). All animal care and experimental procedures complied with the National Institute of Health Guidelines for the Care and Use of Laboratory Animals and were approved by the Animal Ethics Committee of Chengdu University of TCM. Animal studies are reported in compliance with the ARRIVE guidelines [30].

### Moxibustion intervention

In this study, ST36 acupoint (also named Zusanli), located on the posterior lateral side of the knee joint, about 5 mm below the fibula head) was applied to reveal the analgesic effect of moxibustion on chronic pain in that it was proved that acupuncture (needle stimulation) or moxibustion at ST36 generated anti-nociceptive effect in rodent models of chronic pain [14, 20, 22, 26, 27, 31]. Based on the clinical practice of moxibustion to the pain management [32], we set up 30-min treatment duration to the right ST36 with a lighted moxa stick (diameter 4 mm, length 120 mm, made specially for experiment animals by Nanyang Hanyi moxa LLC, Henan, China) (Fig. 1) on the fifth day after CFA injection. During the intervention of moxibustion, the lighted tip of moxa stick was controlled at approximately 1 cm away from ST36.

### Extraction of microdialysates from acupoints

First of all, the microdialysis probe (CMA 20, CMA, Sweden) was implanted into ST36 acupoint under 2% isoflurane anesthesia. After equilibrium for 1.5 h by perfused with Ringer’s solution at a rate of 2 μl per min, the microdialysates were collected on ice over a 30-min period (30 μl) and were immediately frozen at − 80 °C. The ATP inhibitor ARL67156 (Sigma) and adenosine transport inhibitor nitrobenzylthioinosine (Sigma) were added to reduce the degradation ATP and ADO, respectively.

### HPLC analysis of purines

The high-performance liquid chromatography (HPLC, Agilent 1260 Infinity) analysis was applied to measure the concentration of released purine in the interstitial fluid of ST36 acupoints. Chromatographic separation was achieved by using a reverse-phase column (Ultra IBD, LACC-9175565, 5 μm, 150 mm × 4.6 mm, Restek). For measurements of microdialysates, we used a mobile phase consisting of 97.5% ammonium acetate (20 mM), 2.5% methanol, pH 5.8, adjusted by Sartorius PB-10. The flow rate was maintained at 1 ml/min. Daily calibration curves were prepared by a four-point standard (10, 5, 2.5 or 0.5 μM) of ATP, ADP, AMP, and ADO, respectively. Eluted purines were detected at 260 nm by ultraviolet detector, and the chromatographic peaks were integrated using Agilent 1260 Infinity software.

### Injection of ATPase and ARL67156

Before moxibustion intervention, 50 μl of ATPase (125 μg/ml, 250 μg/ml, 500 μg/ml, Sigma) or ARL67156 (62.5 μg/ml, 125 μg/ml, 250 μg/ml, Sigma) was injected (intramuscular injection) into ST36 acupoint. ATPase, known as adenosine triphosphatase, is an enzyme that catalyzes the hydrolysis of ATP to ADP and free phosphate ions. It can promote ATP degradation. ARL67156 (N^6^-diethyl-β, γ-dibromomethylene-ATP trisodium salt hydrate) is a nonspecific ecto-ATPase inhibitor. It was firstly described as the ecto-ATPase inhibitor in 1995 [33] and demonstrated to inhibit ecto-ATPase activity in various tissues of rats [34–37].

### Behavioral assessment

Mechanical allodynia was evaluated using repeated stimulations with a Von Frey filament exerting 2 g of force onto the plantar surface. The percentage of negative responses of a total of ten trials was calculated for each rat. To avoid conditioning to stimulation, we interposed a 5-s rest period. Behavioral assessment was performed before and 5 days postinjection of CFA, and when moxibustion intervention end (last for 2 h with intervals of 30 min).

### Data analysis

All behavioral data were expressed as means ± SEM of *n* observations, where *n* means number of animals per group. The statistical test was performed by repeated measures ANOVA to determine the overall effect of treatment and time; for post hoc analysis, Scheffe multiple comparison test was performed to determine significant differences among groups (SPSS software). A value of *P* < 0.05 was considered to be statistically significant.

## Results

### Moxibustion could trigger local anti-nociceptive effect

**Fig. 2:**
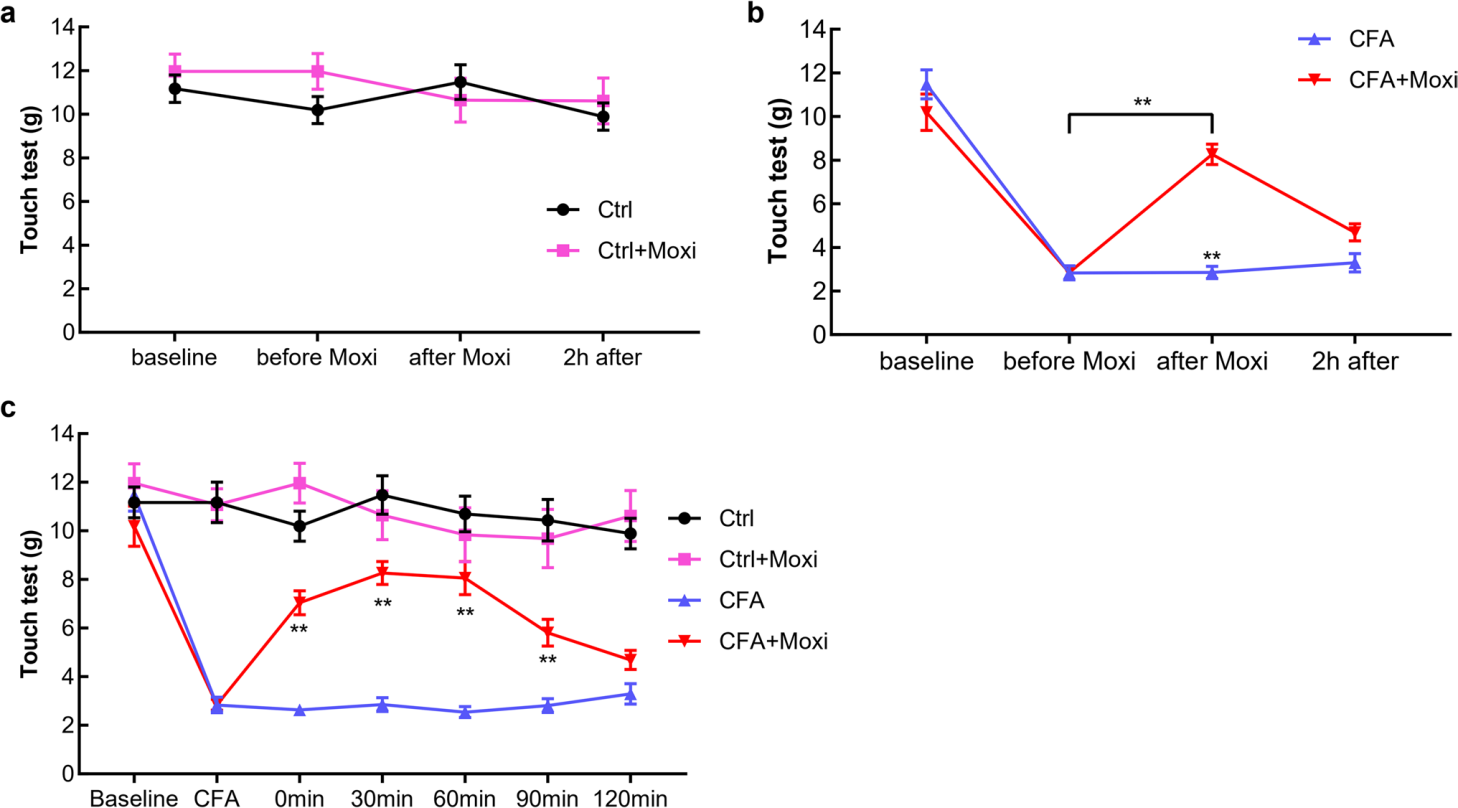
Local anti-nociceptive effects of moxibustion on CFA induced pain model. Moxibustion did not cause obvious changes of pain threshold in control rat (**a**) but reduced sensitivity to mechanical stimulation in CFA + moxibustion group suffering from inflammatory pain after injection of CFA in the right paw (**b**) (**P < 0.01, compared with CFA or before moxibustion). Pain threshold of all rats was evaluated at 0, 30, 90, and 120 min after moxibustion (**c**) (**P < 0.01;compared with CFA). Ctrl control, Moxi moxibustion; *n* = 10 per group

Following injection of CFA, the rat developed decreased mechanical allodynia to innocuous stimulation with Von Frey filaments of the ipsilateral paw peaking at day 5. Administration of the moxibustion at the ST36 evoked increase of touch threshold in CFA + moxibustion group. The pain threshold improved from 2.847 ± 0.229 g to 8.268 ± 0.468 g (*P* < 0.01); however, no significant changes took place in CFA group (*P* > 0.05) (Fig. 2b). Similarly, no significant changes in pain threshold were found between control and moxibustion group (Fig. 2a). After moxibustion, the increased pain threshold was obviously and lasted for 90 min The analgesic effect of moxibustion come to an end 2 h post-moxibustion (Fig. 2c).

### High increased ATP release in response to moxibustion at ST36 acupoint

**Fig. 3:**
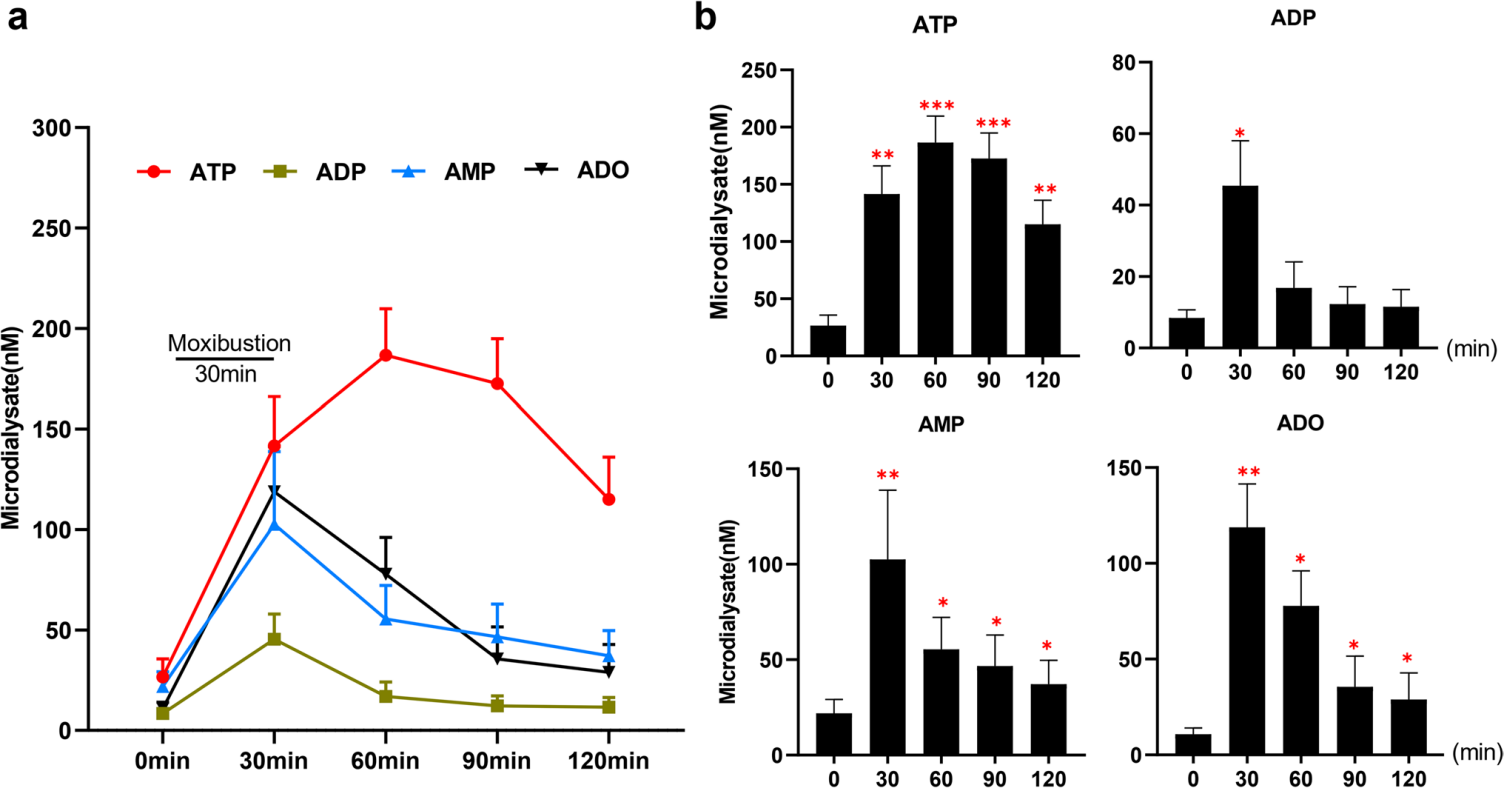
Moxibustion triggers an increase in the extracellular concentration of ATP, ADP, AMP, and ADO. (**a**) Time course of purine release in response to moxibustion. (**b**) Histogram summarizing the mean concentrations of ATP, ADP, AMP, and ADO during baseline non-stimulated conditions, as well as during and following moxibustion (*P < 0.05, **P < 0.01, ***P < 0.001 paired *t* test compared to 0 min, *n* = 6 per group)

To determine which purines are involved in the anti-nociceptive effects of moxibustion, we detected the extracellular concentration of ATP, ADP, AMP, and ADO in ST36 acupoint by HPLC with ultraviolet absorbance before, during, and after moxibustion (Fig. 3a and b). At baseline, the concentrations of ATP, ADP, AMP, and ADO were in the low nanomolar range. After moxibustion intervention for 30 min, the concentrations of 4 purines ATP, ADP, AMP, and ADO were obviously increased, compared to their baseline (*P* < 0.01, *P* < 0.05,* P* < 0. 01,* P* < 0.01). At that 30 min timepoint (moxibustion finished), ADP, AMP, and ADO reached the concentrations peaks, but they decreased obviously at 60 min and closed to their baseline at 90 min or 120 min. The concentration change trends of ADP, AMP, and ADO were similar to the trend in acupuncture analgesia in previous studies [14], but ATP was different. In our study, ATP was featured by an increase trend from 0 to 60 min and reached peak at 60 min with a ~ sevenfold (186.62 ± 23.12 nM) level compared to baseline (*P* < 0.001). Though ATP concentration slightly reduced from 60 to 120 min, it was still much higher than other purine members ADP, AMP, and ADO. This result indicated that ATP might have played an important role in moxibustion-induced analgesia.

### Acceleration of ATP metabolism alleviates moxibustion analgesic effect

**Fig. 4:**
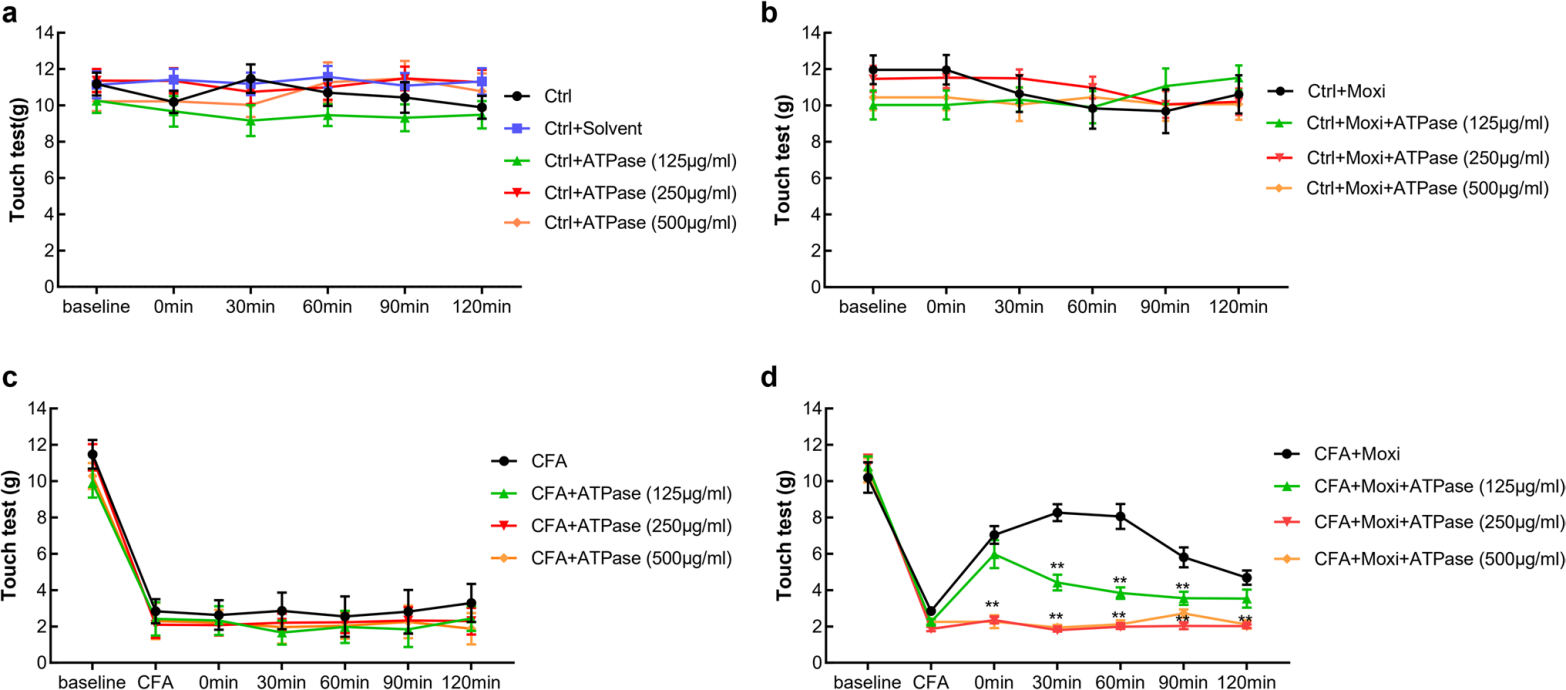
Effect of ATPase injection (125 μg/ml, 250 μg/ml, 500 μg/ml) on moxibustion-induced analgesia. Comparison of the effect of ATPase on mechanical allodynia in every group. CFA was administered in the right paw at day 0. The moxibustion was implemented into the right Zusanli point (ST36) at day 4 (*n* = 10 per group)

We then asked whether that ATPase (a readily available enzyme that breaks down ATP) would diminish or alleviated the analgesic benefits of moxibustion in inflammatory pain. Application of three different concentrations of ATPase (125 μg/ml, 250 μg/ml, 500 μg/ml) to the rats with normal condition (Fig. 4a) did not cause obvious changes of pain threshold in moxibustion intervention group (Fig. 4b) or CFA-induced inflammatory pain group (Fig. 4c). However, administration of ATPase in the right ST36 to the CFA-induced inflammatory pain rats receiving moxibustion therapy sharply reduced moxibustion-induced analgesic effect (Fig. 4d). It showed that 250 μg/ml and 500 μg/ml of ATPase injection had similar effect on reducing the analgesic effect induced by moxibustion, but both of them generated more impact than that 125 μg/ml of ATPase injection (Figs. 4d and 6a, b).

### Inhibition of ATP metabolism enhances moxibustion analgesic effect

**Fig. 5:**
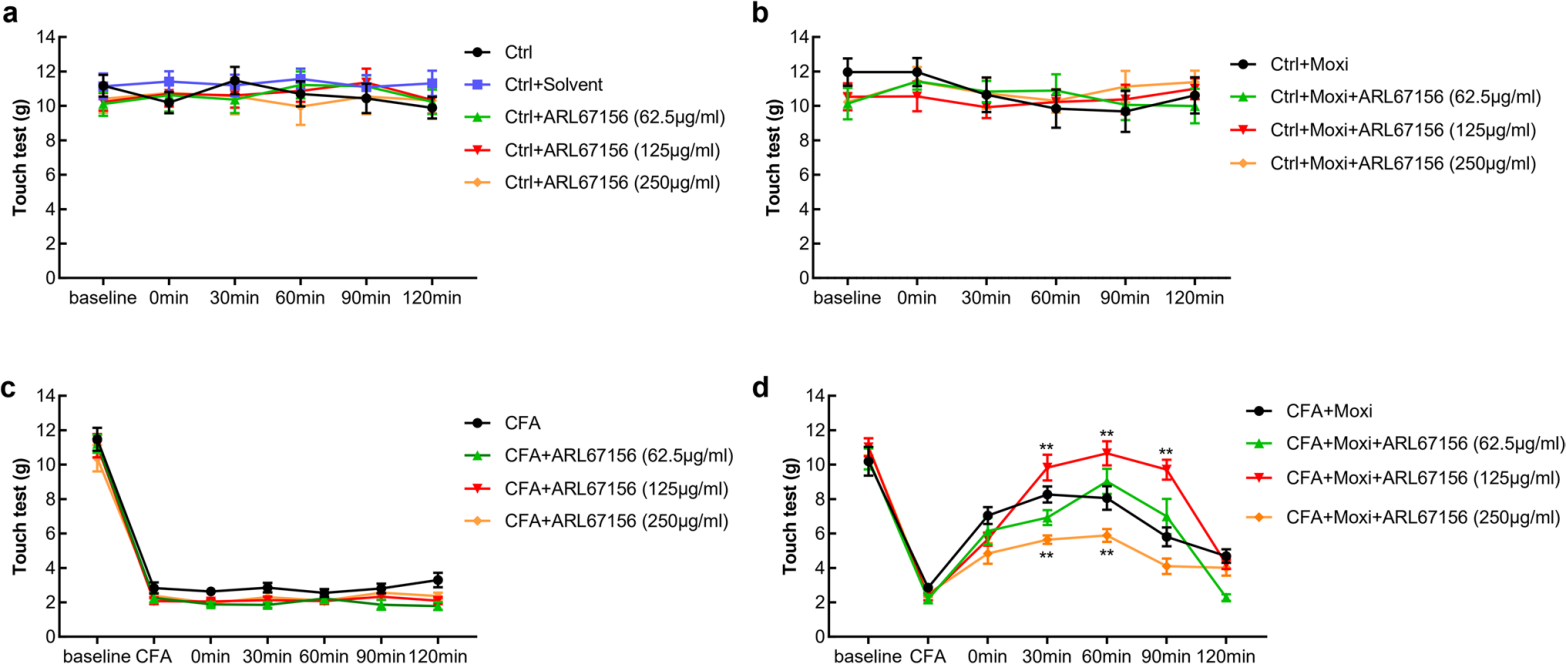
Effect of ARL67156 injection (62.5 μg/ml, 125 μg/ml, 250 μg/ml) on moxibustion-induced analgesia. Comparison of the effect of ARL67156 on mechanical allodynia in every group. CFA was administered in the right paw at day 0. Moxibustion was applied at the right Zusanli point (ST36) at day 4 (*n* = 10 per group)

**Fig. 6:**
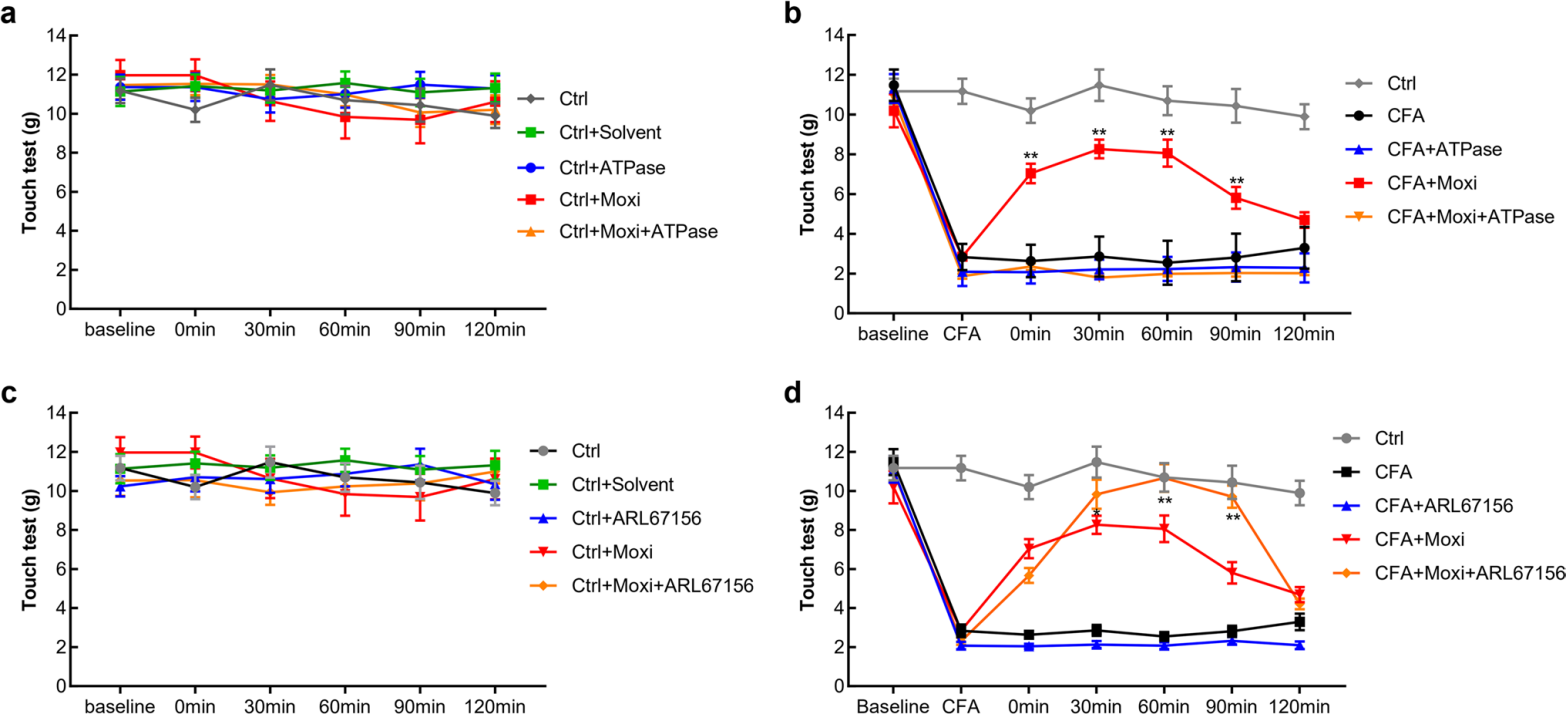
Effect of ATPase (250 μg/ml) and ARL67156 (125 μg/ml) injection on moxibustion-induced analgesia (*n* = 10 per group)

In this study, we also applied three different concentrations (62.5 μg/ml, 125 μg/ml, 250 μg/ml) of ARL67156 to inhibit ATP degradation in order to clarify the role of local ATP in moxibustion-induced analgesia. The data indicated that application of three different concentrations of ARL67156 to the rats with normal condition (Fig. 5a) did not lead to marked changes of pain threshold in moxibustion intervention group (Fig. 5b) or CFA-induced inflammatory pain group (Fig. 5c). And administration of 62.5 μg/ml ARL67156 in the right ST36 to the CFA-induced inflammatory pain rats receiving moxibustion therapy did not cause obvious changes of moxibustion-induced analgesia (Fig. 5d). Injection of 250 μg/ml ARL67156 reduced the analgesic effect induced by moxibustion; however, injection of 125 μg/ml ARL67156 increased the effect of moxibustion-induced analgesia (Figs. 5d and 6c, d).

## Discussion

Moxibustion, as well as acupuncture treatment, has been identified to generate analgesic effect on acidic and purinergically initiated nociceptive hypersensitivity [21] and visceral hyperalgesia [29]. In this study, we firstly found that moxibustion at ST36 is also a painkiller in animal inflammatory pain model. In view of purinergic signalling has close relationship with various pain and the growing evidence for purinergic signalling, especially adenosine, could trigger analgesic effect induced by needle acupuncture [14–22], we wondered if purinergic signalling participates in moxibustion-induced analgesia. Here, we found that moxibustion could trigger high level of extracellular ATP release at ST36, and the time course of ATP release in ST36 acupoint was consistent with moxibustion-induced analgesia effect, which indicated that purinergic ATP might have played a notable role in moxibustion-induced local anti-nociceptive effect. In order to confirm the role of ATP in moxibustion analgesia, we used chemical drugs (ATPase and ARL67156) to speed up or block ATP degeneration at ST36; then, we found that when ATPase was injected into ST36, the analgesic effect of moxibustion did not happen, while it was enhanced when ARL67156 was injected into ST36. Taken together, we would like to propose that the local ATP at ST36 would facilitate to moxibustion-induced analgesia. The reason would be that the locally released ATP activated by the heat [38, 39] generated from moxibustion intervention was speeded up to break down. On the contrary, application of ATPase or ARL67156 to ST36 acupoint of the rats with normal condition did not cause obvious changes of pain threshold in moxibustion group or inflammatory pain group; however, administration of ATPase or ARL67156 in ST36 to inflammatory pain rats receiving moxibustion therapy sharply reduced or enhanced moxibustion-induced analgesic effect. As mentioned above, both moxibustion and acupuncture could trigger analgesia effect and the mechanism get involved in purinergic signalling. Why ATP plays an important role in moxibustion-induced analgesia, but ADO is responsible for acupuncture analgesia? It is a question worthy of our in-depth exploration. It might be because moxibustion and acupuncture are two different stimulations on analgesia acupoint. Moxibustion mainly works through warm stimulation, while acupuncture mainly works by mechanical stimulation. Maybe different purines have different response sensitivity to different stimulation.

Another question is raised by the role of use of ATPase or ARL67156 in pain. If ATP released from the local acupoint and more ATP was kept, why the outcome is analgesic instead of nociceptive effect? Previous study indicated that delivery of ATP to the forearm skin of volunteers by iontophoresis caused higher pain rating [40] although intravenous ATP would generate pain relief [41, 42]. Maybe one potential explanation would be that moxibustion is an alternative therapy in which the heat is produced by burning herbs which caused release of ATP and active P2X3 receptor to affect the thermal transduction [43].

In a word, current data implied that purinergic ATP at the location of ST36 acupoint is a potentially beneficial factor for moxibustion-induced analgesia.

## Data Availability

The datasets generated during and/or analyzed during the current study are available from the corresponding author on reasonable request.
